# Clinical and Molecular Epidemiology of *Haemophilus influenzae* Causing Invasive Disease in Adult Patients

**DOI:** 10.1371/journal.pone.0112711

**Published:** 2014-11-07

**Authors:** Carmen Puig, Imma Grau, Sara Marti, Fe Tubau, Laura Calatayud, Roman Pallares, Josefina Liñares, Carmen Ardanuy

**Affiliations:** 1 Department of Microbiology, Hospital Universitari de Bellvitge, Universitat de Barcelona-IDIBELL, Barcelona, Spain; 2 Department of Infectious Diseases, Hospital Universitari de Bellvitge, Universitat de Barcelona-IDIBELL, Barcelona, Spain; 3 CIBER de Enfermedades Respiratorias (CIBERes), ISCIII, Madrid, Spain; University of Cambridge, United Kingdom

## Abstract

**Objectives:**

The epidemiology of invasive *Haemophilus influenzae* (Hi) has changed since the introduction of the Hi type b (Hib) vaccine. The aim of this study was to analyze the clinical and molecular epidemiology of Hi invasive disease in adults.

**Methods:**

Clinical data of the 82 patients with Hi invasive infections were analyzed. Antimicrobial susceptibility, serotyping, and genotyping were studied (2008–2013).

**Results:**

Men accounted for 63.4% of patients (whose mean age was 64.3 years). The most frequent comorbidities were immunosuppressive therapy (34.1%), malignancy (31.7%), diabetes, and COPD (both 22%). The 30-day mortality rate was 20.7%. The majority of the strains (84.3%) were nontypeable (NTHi) and serotype f was the most prevalent serotype in the capsulated strains. The highest antimicrobial resistance was for cotrimoxazole (27.1%) and ampicillin (14.3%). Twenty-three isolates (32.9%) had amino acid changes in the PBP3 involved in resistance. Capsulated strains were clonal and belonged to clonal complexes 6 (serotype b), 124 (serotype f), and 18 (serotype e), whereas NTHi were genetically diverse.

**Conclusions:**

Invasive Hi disease occurred mainly in elderly and those with underlying conditions, and it was associated with a high mortality rate. NTHi were the most common cause of invasive disease and showed high genetic diversity.

## Introduction

*Haemophilus influenzae* is a human-restricted pathogen that forms part of the normal nasopharyngeal microbiota. The presence or absence of a polysaccharide capsule divides this bacterium into two different groups [1], [2]. The variability of capsular polysaccharide means that encapsulated *H. influenzae* strains are classified into six serotypes, labeled a–f. Before the introduction of the conjugate vaccine against *H. influenzae* type b (Hib), Hib was the major serotype responsible for invasive infections in infants and young children, with meningoencephalitis as the most common clinical manifestation [1], [3], [4]. With the prevention provided by vaccination, colonization rates and invasive infections in children have been considerably reduced [1], [3]–[6]. Non-capsulated strains, also known as nontypeable *H. influenzae* (NTHi), frequently cause respiratory infections such as otitis media in children and exacerbations of chronic respiratory diseases and community-acquired pneumonia in fragile adult populations [1], [2], [7]–[9]. Notably, since introduction of the Hib vaccine a strain replacement has been observed in invasive infections and nontypeable strains have become predominant among cases of invasive *H. influenzae* disease in adults [10], [11].

The main aims of this study were to analyze the demographic and clinical characteristics of adult patients with invasive *H. influenzae* infections and to determine the antimicrobial resistance and molecular epidemiology of these invasive strains.

## Materials and Methods

### Ethical Statement

This study has been revised and approved for its publication by the Clinical Research Ethics Committee of Bellvitge University Hospital (PR223/14). Written informed consent was considered not necessary for the study, as it was a retrospective analysis of our usual everyday work. The data of the patients were anonymized for the purposes of this analysis. The confidential information of the patients was protected according to national normative.

### Study design and clinical data

A six-year laboratory-based study (2008–2013) was conducted at the Bellvitge University Hospital, a tertiary care center for adult patients located in the south of Barcelona (Spain), the aim being to analyze the epidemiology of invasive *H. influenzae*. Invasive *H. influenzae* were defined as the isolation of Hi from blood, cerebrospinal fluid (CSF), or pleural fluid with clinical symptoms in the patient. The denominator used to estimate incidence was the number of persons by age group per year recorded in the public database hosted on the website of the Official Statistics Office of Catalonia (http://www.idescat.cat). Clinical and demographical data were retrieved from a prospective protocol of bacteremia cases recorded at our institution; in those patients without bacteremia their clinical records were reviewed.

### Bacterial Strains

Invasive *H. influenzae* strains collected from sterile sites in our laboratory were stored at −80°C. Isolates were identified by mass spectrometry using a MALDI-TOF Biotyper version 3.0 (Bruker). Differentiation between *H. influenzae* and *H. haemolyticus* was performed by the detection of *fuc*K, *iga*, and *lgt*C genes, using a previously described methodology [12]. Isolates with a positive detection for all three genes were considered *H. influenzae*.

### Capsule Typing and Antimicrobial Susceptibility Testing

Capsular serotype was determined by PCR using primers and conditions described elsewhere [13]. Antimicrobial susceptibility was tested in all 82 isolates by disk diffusion as a part of the normal laboratory routine, following CLSI recommendations. In the 70 available isolates, minimal inhibitory concentration (MIC) was tested by the microdilution method using commercial panels (STRHAE2; Sensititre, West Sussex, England) and following Clinical Laboratory Standards Institute (CLSI) recommendations [14], [15]. β-lactamase activity was screened using the chromogenic cephalosporin method (nitrocefin disks, BD, Madrid, Spain). Identification of β-lactamase type was performed by PCR on all the positive β-lactamase isolates using previously described primers and conditions [16].

### PPB3 Sequencing and Genotype Definition for Ampicillin Resistance

An internal region of the *fts*I gene (796-1741 pb) was amplified by PCR and sequenced as previously described [17]. In accordance with previous descriptions [18], [19], *H. influenzae* were classified into four ampicillin-resistant genotypes: β-lactamase negative ampicillin susceptible (gBLNAS), strains without a detectable resistance mechanism; β-lactamase negative ampicillin resistant (gBLNAR), strains which presented mutations in the *fts*I gene; β-lactamase positive ampicillin resistant (gBLPAR), strains producing β-lactamase; and β-lactamase positive amoxicillin/clavulanic acid resistant (gBLPACR), strains which presented both resistance mechanisms (β-lactamase production and mutations in the *fts*I gene).

### Molecular Typing

Genomic DNA was digested with *Sma*I and the fragments were separated by pulsed-field gel electrophoresis (PFGE), as reported previously [9]. PFGE band patterns were analyzed using the Fingerprinting II Software 3.0 (BioRad). The similarity of the PFGE banding patterns was estimated with the Dice coefficient, setting the optimization and tolerance at 1%. Isolates with ≥80% relatedness were considered highly genetically related [20]. Multilocus sequence typing (MLST) was performed by DNA sequencing of internal fragments of seven housekeeping genes (*adk*, *atp*G, *frd*B, *fuc*k, *mdh*, *pgi*, and *rec*A), as previously described [21]. Allele number and sequence types (ST) were assigned using the *H. influenzae* MLST website (http://haemophilus.mlst.net).

### Statistical Analyses

Statistical analyses were performed using GraphPad Prism version 4, using Chi-square or Fisher’s exact tests, when appropriate, with P<.05 being considered significant.

## Results

### Clinical characteristics

During the period 2008 to 2013 a total of 3433 *H. influenzae* were isolated from adult patients in our hospital. Of these, 82 isolates (2.4%) caused invasive *H. influenzae* infection in 82 patients. The overall incidence for Hi invasive disease among adults in our area during the study period was 2.12 episodes per 100,000 population. By age group the incidence of invasive disease was higher among those aged 65 or older than among people ≤64 years (6.8/100,000 *vs*. 1.1/100,000; p<0.01). We observed no significant change in the incidence of invasive disease over the study period, neither overall nor by age group or serotype.

Table 1 shows the demographics, clinical characteristics, and underlying conditions for the 82 patients with invasive *H. influenzae* infection. Fifty-two (63.1%) cases occurred in men and the mean age of patients was 64.3 years. Most cases were community-acquired, and pneumonia was the most frequent type of infection (59.8%) The most common comorbidities were immunosuppressive conditions, malignancies, diabetes, chronic obstructive pulmonary disease (COPD), and heart disease (Table 1). In general, older patients had higher rates of underlying conditions than did those ≤64 years, especially for COPD (34.0% vs. 5.7%, p = 0.002) and heart disease (27.6% vs. 5.7%, p = 0.018). The 30-day mortality was 20.7% (n = 17), with no differences between younger and older adults (p = 0.58).

**Table 1:** Demographic data, clinical characteristics, and underlying conditions of 82 patients with an invasive *H. influenzae* episode during the period 2008–2013.

	Patients (n = 82)
Characteristics [no. (%)]	
Age (mean ± SD); range	64.3±16.1; 21–96
Male sex	52 (63.4)
Acquisition	
Community-acquired	71 (86.6)
Hospital-acquired	11 (13.4)
Source of infection	
Pneumonia/empyema	49 (59.8)
Meningitis	9 (11.0)
Biliary tract infection	9 (11.0)
Primary bacteremia	7 (8.5)
Epiglottitis	2 (2.4)
Others^a^	6 (7.3)
Underlying conditions [no. (%)]	
Immunosuppressive therapy	28 (34.1)
Solid organ malignancy	26 (31.7)
Diabetes	18 (22.0)
COPD	18 (22.0)
Heart disease	15 (18.3)
Chronic liver disease	11 (13.4)
Hematologic malignancy^b^	9 (11.0)
Cerebrovascular disease	5 (6.1)
Organ transplant^c^	4 (4.9)
HIV	2 (2.4)
Others^d^	10 (12.2)
Shock	18 (22.0)
Neutropenia	8 (9.8)
Mortality	
<30 days	17 (20.7)

a Facial cellulites, endometritis, liver abscess, and urinary-tract infection (n = 1, 1.2% each), and peritonitis (n = 2, 2.4%).

b Leukemia (n = 3, 3.6%), lymphoma (n = 1, 1.2%), and myeloma (n = 5, 6.1%).

c Bone marrow transplant (n = 1, 1.2%), kidney transplant (n = 1, 1.2%), and liver transplantation (n = 2, 2.4%).

d Cerebrospinal fluid fistula (n = 2, 2.4%), renal failure, autoimmune disease, and head trauma (n = 1, 1.2% each).

### Invasive *H. influenzae*: sample origin and serotypes

The source of strains in the 82 invasive Hi cases was: blood (n = 70), CSF (n = 3), pleural fluid (n = 2), blood plus CSF (n = 5), and blood plus pleural fluid (n = 2). Serotypes of isolates causing meningitis were NTHi (n = 6) and Hif (n = 1), with one isolate being unavailable (n = 1).

**Figure 1:**
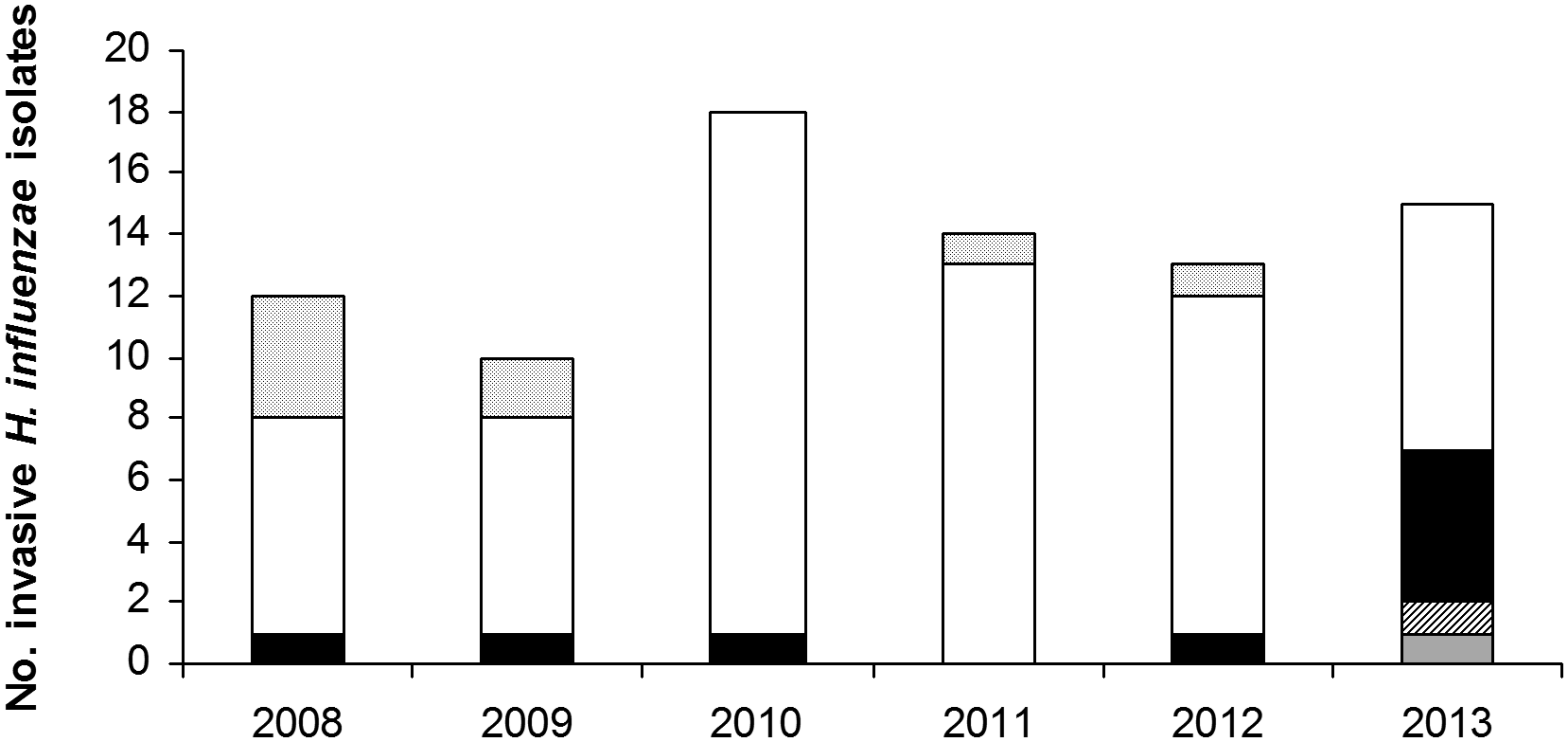
Distribution of 82 invasive *H. influenzae* isolated from adult patients (2008–2013). White bar: nontypeable *H. influenzae*. Black bar: *H. influenzae* serotype f. Dotted bar: no available isolated for serotyping. Grey bar: *H. influenzae* serotype b. Lined bar: *H. influenzae* serotype e.

Unfortunately, only isolates from 70 cases (70/82, 85.37%) were viable and available for microbiological studies. The majority of these isolates were NTHi (n = 59, 84.3%), and the frequency of capsulated isolates was low (n = 11, 15.7%). Among 11 capsulated strains, 9 were serotype f (Hif), 1 serotype b (Hib), and 1 serotype e (Hie) (Figure 1). Encapsulated *H. influenzae* were mainly isolated from blood (n = 9, 81.8%). Both Hib and Hie strains were isolated from patients with pneumonia. The foci of infection of Hif cases were pneumonia (n = 3), epiglottitis (n = 1), meningitis (n = 1), peritonitis (n = 1), facial cellulites (n = 1), and biliary tract infection (n = 1).

### Antimicrobial susceptibility

All 82 isolates tested by disk diffusion presented fully susceptibility to amoxicillin/clavulanic acid, cefotaxime, ceftriaxone, chloramphenicol, tetracycline, and ciprofloxacin. On the other hand, 23% of the isolates (n = 19) were resistant to cotrimoxazole and 8.5% (n = 7) were resistant to ampicillin due to β-lactamase production. Antimicrobial susceptibility of the 70 available isolates was tested by microdilution. All tested isolates were fully susceptible to amoxicillin/clavulanic acid, cefepime, cefotaxime, ceftriaxone, imipenem, chloramphenicol, tetracycline, and ciprofloxacin (Table 2). Capsulated isolates were fully susceptible to all tested antibiotics, with the exception of one strain that was resistant to rifampin. By contrast, NTHi isolates presented higher resistance rates to cotrimoxazole (27.1%), azithromycin (1.4%), and cefuroxime (1.4%) (Table 2). Seven NTHi isolates (10%) were ampicillin resistant: six (6/70, 8.6%) due to β-lactamase production (MIC>4 mg/L) and one due to a modified PBP3 (MIC = 4 mg/L). In addition, three strains (3/70, 4.3%) presented reduced ampicillin susceptibility (MIC = 2 mg/L). All six β-lactamase producers presented the *bla*_TEM-1_ gene.

**Table 2:** Antimicrobial susceptibility of 70 invasive *H. influenzae*.

	MIC_50_	MIC_90_	Range	% S	% I	% R
Antimicrobial	(mg/L)	(mg/L)	(mg/L)			
Ampicillin^a^	0.25	2	≤0.12–>4	85.7	4.3	10.0
Amoxicillin/clavulanicacid^b^	≤0.5	2	≤0.5–4	100		
Cefuroxime	1	2	≤0.5–8	98.6	1.4	
Cefepime	≤0.25	≤0.25	≤0.25–0.25	100		
Cefotaxime	≤0.06	≤0.06	≤0.06–0.06	100		
Ceftriaxone	≤0.12	≤0.12	≤0.12	100		
Imipenem	0.5	1	≤0.12–2	100		
Chloramphenicol	≤1	≤1	≤1	100		
Tetracycline	≤1	2	≤1–2	100		
Ciprofloxacin	≤0.03	≤0.03	≤0.03	100		
Cotrimoxazole^c^	≤0.5	>2	≤0.5–>2	72.9	1.4	25.7
Azithromycin	1	2	≤0.12–>4	98.6		1.4

a β-lactamase production: 8.6% (n = 6).

b The ratio of amoxicillin/clavulanic acid was 2∶1.

c The ratio of cotrimoxazole was 1∶19.

In order to determine the mutations in PBP3 the transpeptidase domain of the *fts*I gene was sequenced in all the isolates. Thirty-four isolates (48.6%) presented mutations in the *fts*I gene (Table 3). The observed mutations allow us to classify the strains into groups I and II, in accordance with Dabernat *et al*. [17]. The most frequent substitutions were those which were classified as group II (22/34, 64.7%). No isolates were observed in subgroup IId or in groups III and III-like. Additionally, eight isolates (23.5%) presented mutations in the *fts*I gene, none of which were at the positions which defined the groups. For this reason, they were considered gBLNAS and classified into the miscellaneous group. All these eight isolates presented similar ampicillin MIC to susceptible strains (≤0.5 mg/L), suggesting that these mutations were not involved in decreased β-lactam susceptibility (Table 3).

**Table 3:** Amino acid substitutions in PBP3 among 70 invasive *H. influenzae* strains.

BLNAR/BLPACR Genotype	Noisolates	Amino acid substitutions													MIC (mg/L)		BL^a^
		Ile 348	Asp350	Ala368	Met377	Met391	Ala437	Gly490	Ala502	Val509	Arg517	Asn526	Ala530	Phe531	AMP	AMC	
No changes^b^	33														≤0.12	≤0.5	–
	3														>4	≤0.5	+
I	3										His				0.5	1	–
	1								Thr		His						–
IIa	1											Lys			1	2	–
	2		Asn					Glu				Lys	Ser		1	1–2	–
IIb	1								Val			Lys			0.5	1	–
	6		Asn		Ile				Val			Lys			0.5–4	1–4	–
	1		Asn		Ile				Val			Lys			>4	4	+
	2		Asn		Ile			Glu	Val			Lys			0.5–1	1–2	–
	1		Asn		Ile			Glu	Val			Lys			>4	4	+
IIc	2								Thr			Lys			1–2	2–4	–
	1								Thr			Lys			>4	4	+
	5		Asn						Thr			Lys			1–2	2–4	–
Miscellaneous^c^	1	Val													0.5	1	–
	1			Thr											≤0.12	≤0.5	–
	1		Asn												0.12	≤0.5	–
	2									Ile					0.25	≤0.5	–
	1													Leu	0.25	≤0.5	–
	2						Ser								0.25	≤0.5	–

a BL: Beta-lactamase production: + (positive); – (negative).

b Isolates without amino acid changes in PBP3 (gBLNAS).

c Isolates grouped in the miscellaneous group were not gBLNAR.

### Molecular epidemiology

Molecular typing by PFGE revealed 50 different patterns. Fourteen clusters grouped between two and seven related isolates, and 36 patterns were genotypically unique. Hif isolates were grouped into two related clusters, one with seven isolates and the other with two. The other 12 clusters grouped two NTHi isolates each.

Molecular typing by MLST showed high genetic variability among 59 NTHi isolates, which had 51 different sequence types (STs). After eBURST analysis, NTHi STs were distributed into three clonal groups, along with 45 singletons. Clonal group 1 grouped ST103 and ST134, with one isolate each. Group 2 was formed by three isolates, with ST3 (n = 1) and ST367 (n = 2). Finally, clonal group 3 comprised ST14 (n = 1) and a single locus variant (SLV, n = 1). Among the 45 singletons the most frequent ST was ST57, with three isolates. eBURST analysis of all the STs from NTHi isolates published in the MLST database revealed that 75.7% (n = 53) of our invasive isolates belonged to different clonal complexes (CC) defined by the analysis. Twenty-three isolates belonged to the five most prevalent CC among NTHi: CC1, CC3 (n = 5 each), CC41, CC57, and CC472 (n = 3 each).

Capsulated strains, by contrast, were genetically related. All type f isolates belonged to CC124, formed by ST124 (n = 7) and two single locus variants (*rec*A5 and *rec*A43). The type b strain belonged to CC6 (SLV ST6; *rec*A15), while the Hie strain belonged to ST18.

## Discussion

The epidemiology of invasive *H. influenzae* has changed since the introduction of the Hib conjugate vaccine for children, with nontypeable strains being the most frequent etiological agent in most cases of invasive Hi disease in adults. The incidence rate observed among our adults (2.12/100,000) is similar to that reported in the USA and in Europe [6], [22], [23]. As documented in other studies the incidence of invasive Hi disease increased with age (6.8/100,000 in patients ≥65 years old) [6], [23].

In the pre-vaccine era, Hib was the most important cause of invasive disease (mainly meningitis) in healthy children under 5 years of age [1], [4], [22]. In the United States the pre-vaccine incidence of invasive disease in adults was 1.7 cases per 100,000 persons, with Hib being responsible for 50% of invasive diseases due to *H. influenzae* [11]. In the United Kingdom the incidence of Hib infection in adults was low (9%) and it was assumed to be a consequence of transmission from children [24]. Since the widespread childhood immunization program in the 1990s, Hib infection has decreased considerably worldwide [4]. The percentage of Hib infection cases in adults varies depending on the region. In Illinois, for example, 17.7% of cases were Hib, whereas in Utah the figure was 9% [6], [11]. Only one of our isolates was identified as Hib (1.4%), a lower percentage than previously reported (4.92%) in another Spanish study [25]. Concerning non-b capsulated strains, Hif is currently the most frequent serotype causing invasive disease [1], [5], [11], [25]–[27]. However, despite the fact that in our hospital the number of invasive *H. influenzae* isolated from 2008–2013 remained stable, an increment in non-b capsulated strains was observed in 2013, when the number of capsulated isolates doubled due to an increase in serotype f.

During the pre-vaccination era, NTHi were not a frequent cause of invasive disease, even though they had been considered an important respiratory pathogen in adults [8]. However, a strain shift has been observed since vaccination, with NTHi being the strains most frequently responsible for invasive infections in adults [1]. NTHi were the most common cause of invasive Hi infection in adults in Illinois (34%), Utah (43%), Manitoba (57%), and Sweden [6], [11], [26], [27]. A recent publication in Spain reported 62% of NTHi among invasive isolates from adults (2004–2009) [25]. In our study, which analyzed invasive isolates from 2008 to 2013, 85.7% of strains were identified as NTHi, following the trend observed in other studies and adding to previously published data on more recently isolated strains.

As reported in other studies [3], [6], [11], most invasive *H. influenzae* disease infections occurred in older adults (n = 47 patients were ≥65 years old) and in those with underlying conditions. Increased life expectancy and the growing number of patients with underlying conditions may account for the high proportion of invasive Hi disease found in the present study. Pneumonia was the most common type of infection caused by invasive *H. influenzae* in adults, as reported elsewhere [10], [22], [24],[28]. In line with other studies [3], [6], [11], [28], [29], our patients with invasive *H. influenzae* infection showed a high mortality rate, although again this could be associated with age and underlying conditions. Nevertheless, we have to acknowledge the small number of cases identified as a limitation of our study.

Following the clinical and epidemiological evaluation of samples the study aimed to investigate the antimicrobial resistance of invasive *H. influenzae*. Traditionally, the most common mechanism of β-lactam resistance in *H. influenzae* has been β-lactamase production, although this production has decreased over time [30]–[32]. The percentage of β-lactamase in invasive *H. influenzae* varies depending on the study, ranging from 10–24% [3], [25], [26], [32]–[38]. In our study the percentage of β-lactamase production was 8.6%, in line with published data although lower than the figures (16.9% and 24.2%) reported in two previous studies about invasive *H. influenzae* performed in 1999–2000 and 2004–2009 in Spain [3], [25]. β-lactam resistance due to alterations in PBP3 has also been reported worldwide [18], [26], [30], [39], [40]. In the present study, 32.9% of isolates were considered gBLNAR, with ampicillin MIC of 0.5–4 mg/L, presenting relevant mutations in the transpeptidase domain of *fts*I. Although these mutations conferred reduced susceptibility to ampicillin, the isolates in question were not considered resistant according to current CLSI and EUCAST breakpoints, the exception being one strain which presented an ampicillin MIC of 4 mg/L. In our experience, the patients infected by BLNAR strains were successfully treated with amoxicillin/clavulanic acid, cefepime, ceftriaxone, piperacillin/tazobacatam, and quinolones (data not shown). Currently, these strains with altered MICs to β-lactams can be successfully treated with these antibiotics; however, the detection of BLNAR strains in the laboratory could improve the knowledge about the epidemiology of *H. influenzae*. The most common mutations found in invasive isolates were those that classified the strains into group IIb (47.8%), this being consistent with previous data reported by Resman *et al*., Shuel *et al*., and Bajanca *et al*. [26], [33], [37]. By contrast, however, with Spanish data published by García-Cobos *et al*., who found that group IIc was the most common BLNAR genotype in invasive *H. influenzae* (42.4%) [25].

Genotyping by PFGE and MLST showed a high diversity among NTHi strains, which were distributed into three clonal groups and 45 singletons. Despite the high genetic variability observed in NTHi, the majority of isolates were grouped according to the most prevalent clonal complexes defined by eBURST, using all the NTHi published in the MLST database. Capsulated strains, by contrast, were clonally related. These results are consistent with other studies that also described this difference in diversity between NTHi and capsular *H. influenzae* [26], [33], [37]. Moreover, all capsulated strains found in our study belonged to international disseminated global clones. For instance, all nine Hif isolates were grouped in CC124 (ST124 and two single locus variants), which has been identified in the USA and other European countries [25], [33], [37], [38]. Hib and Hie isolates belonged, respectively, to the CC6 and CC18 clones, which have been detected worldwide (http://haemophilus.mlst.net/).

In conclusion, NTHi were the most frequent cause of invasive Hi disease in adults, who frequently presented underlying conditions, and they were associated with a high mortality rate. In our hospital, however, there was an increase in capsular strains, generally serotype f, during 2013. It should also be noted that reduced ampicillin susceptibility was observed in a high percentage of invasive *H. influenzae* due to mutations in PBP3. Despite the reduction in Hib, continuous monitoring of invasive *H. influenzae* infections should be performed, not only because of the recent increase in capsulated non-b strains but also in order to detect changes in the epidemiology of invasive *H. influenzae*.
